# Efficient and Effective Use of Peer Teaching for Medical Student Simulation

**DOI:** 10.5811/westjem.2016.11.32753

**Published:** 2016-11-15

**Authors:** Joseph B. House, Carol H. Choe, Heather L. Wourman, Kristin M. Berg, Jonathan P. Fischer, Sally A. Santen

**Affiliations:** * University of Michigan Medical School, Department of Emergency Medicine, Ann Arbor, Michigan; † University of Michigan Medical School, Department of Pediatrics, Ann Arbor, Michigan; ‡ University of Michigan Medical School, Department of Learning Health Sciences, Ann Arbor, Michigan; § University of Michigan, Department of Health Management and Policy, Ann Arbor, Michigan; ¶ Cooper University Hospital, Department of Medicine, Division of Critical Care Medicine, Camden, New Jersey; || Memorial Hermann Northeast Hospital, Department of Emergency Medicine, Humble, Texas

## Abstract

**Introduction:**

Simulation is increasingly used in medical education, promoting active learning and retention; however, increasing use also requires considerable instructor resources. Simulation may provide a safe environment for students to teach each other, which many will need to do when they enter residency. Along with reinforcing learning and increasing retention, peer teaching could decrease instructor demands. Our objective was to determine the effectiveness of peer-taught simulation compared to physician-led simulation. We hypothesized that peer-taught simulation would lead to equivalent knowledge acquisition when compared to physician-taught sessions and would be viewed positively by participants.

**Method:**

This was a quasi-experimental study in an emergency medicine clerkship. The *control* group was faculty taught. In the peer-taught *intervention* group, students were assigned to teach one of the three simulation-based medical emergency cases. Each student was instructed to master their topic and teach it to their peers using the provided objectives and resource materials. The students were assigned to groups of three, with all three cases represented; students took turns leading their case. Three groups ran simultaneously. During the intervention sessions, one physician was present to monitor the accuracy of learning and to answer questions, while three physicians were required for the control groups. Outcomes compared pre-test and post-test knowledge and student reaction between control and intervention groups.

**Results:**

Both methods led to equally improved knowledge; mean score for the post-test was 75% for both groups (p=0.6) and were viewed positively. Students in the *intervention* group agreed that peer-directed learning was an effective way to learn. However, students in the *control* group scored their simulation experience more favorably.

**Conclusion:**

In general, students’ response to peer teaching was positive, students learned equally well, and found peer-taught sessions to be interactive and beneficial.

## INTRODUCTION

The ability to recognize a patient who requires immediate care, to initiate treatment, and to seek additional support is essential for all graduating medical students.^1^ While clinical education focuses on common clinical presentations and acute management skills, the emphasis on patient safety, billing, and patient satisfaction in recent years has resulted in the marginalization of medical students in the clinical setting.^2^ In addition, ethical questions are raised around the traditional practice of “see one, do one, teach one.”^3^ In order to maintain high-quality education in a safe environment, simulation has become increasingly important in medical education. Simulation has the advantage of introducing students to serious clinical conditions in a standardized and non-threatening manner^2^ without involving actual patients and provides an environment for students to gain practice teaching,^4^–^8^ which also increases their knowledge retention.^9^ The benefits of simulation in medical education have been well documented.^2^,^10^,^11^ However, simulation training requires considerable resources, not the least of which is faculty time required for preparation and delivery. This study aimed to test the feasibility and effectiveness of student peer-taught simulations in an emergency medicine (EM) clerkship. Our hypothesis was that student peer-taught simulation sessions would lead to comparable knowledge acquisition when compared to physician-taught sessions, as students would be on a similar educational level and thus understand the needs of their peers. Students may also feel more comfortable asking their peers questions instead of a physician. Additionally, we hypothesized that the peer-taught participants would view simulation positively.

## METHODS

This was a quasi-experimental design study. The setting was an academic emergency department where EM is a required fourth-year clerkship. The subject population was students rotating through the clerkship from January 2013 – December 2013. The university’s institutional review board reviewed this study and determined it to be exempt.

Students rotating through the ED are required to attend core didactic lectures of basic EM concepts and simulation sessions. These sessions are integrated into the didactic teaching days so students can quickly apply the knowledge. Students were assigned cases on day two of the rotation and presented the cases 1–2 weeks later. Three simulation-based clinical scenarios were developed that are considered high yield for EM, including ACLS algorithms, and could be taught using simulation (Laerdal Little Anne CPR training manikin with a rhythm generator). These cases included management of a basic disease process followed by stabilization of a life-threatening cardiac dysrhythmia. Cases were asthma exacerbation decompensating into supraventricular tachycardia (SVT), acute myocardial infarction developing symptomatic bradycardia, and congestive heart failure leading to ventricular tachycardia.

In 2012, as part of a pilot, EM faculty developed the cases. A group of six fourth-year medical students assisted with the revision of cases and materials along with providing important feedback to improve the process prior to the launch of the study. These students were involved in a pilot test of student-taught simulations to ensure all material was presented and to improve construct validity. Initially, students felt they needed more direction on the peer-taught cases. The instructions were adjusted to improve these aspects.

For the study period, peer-taught simulation (intervention group) and physician-taught simulation (control group) alternated months. Due to not having enough physician volunteers, some months were converted from physician-taught to student-taught and thus there are not equal numbers between the two cohorts.

*Intervention group:* (111 students) At the beginning of the four-week rotation, each student was assigned a case. Each case had a list of objectives, patient encounter summary, outcomes checklist, questions to facilitate debriefing, instructions on how to use the rhythm generator and a list of resources for the topic. 12 Students were encouraged to augment their knowledge of the topic with outside reading.

On the day of simulation, the students were assembled into groups consisting of at least one peer leader for each case. During periods in which the number of students was not a multiple of three, the group had more than one student assigned to the same case; in this situation, the cases were co-taught. To ensure standardized delivery of basic instructional components and to minimize the potential confounding variable effects of multiple instructors, the student peer teachers were given the same set of instructions, the same objectives, and debriefing questions.

The peer teacher was instructed to run the basic medical simulation (e.g., asthma case) for about five minutes before transitioning to the cardiac dysrhythmia (e.g., supraventricular tachycardia). After another five minutes, they would end the session allowing the last five minutes for debriefing and discussion. The simulators were set in a “U” shape with the emergency physician in the middle available to answer questions that were beyond the scope of the peer leader’s knowledge and to monitor teaching and learning of the three groups of students. Each table was given a label “A,” “B,” and “C.” The student who had that case, A, B, or C, presented his/her case. Each group started on a different case, and thus at any one time all three cases were being taught. After each case (15 minutes), the students would rotate, and another peer leader would present his case. As such, each student was a peer teacher for one case, and a peer learner for two. One physician volunteer was required for each session.

*Control Group:* (65 students) Resident or faculty volunteers were provided the cases and objectives 1–2 weeks prior to the simulation session. They were given the same case packets and instructions as the students in regard to five minutes for the medical emergency, five minutes for the dysrhythmia, and five minutes for debriefing, along with objectives and debriefing instructions. After the completion of the case, the students moved to the next case. Three physician volunteers were required for each session, one for each case.

The intervention outcomes were evaluated on two levels. The intervention and control groups were given pre- and post-knowledge tests. The teaching objectives for each case were used for test development. The pre-test was administered on the first day of the rotation, prior to providing the cases to the intervention group. The test was piloted in the fall of 2012 and subsequently revised. Students were also surveyed regarding their attitudes toward peer- versus physician-led teaching on a five-point Likert scale from strongly disagree to strongly agree. Attention was paid to content and response process validity through the instrument design, and also to internal validity (Crohnbach’s alpha for the attitudes outcomes survey was 0.9).

### Statistical Analysis

We obtained descriptive statistics using SPSS 19. The differences in attitudes between the control and peer teaching groups were compared using a Mann-Whitney U. We compared the differences in knowledge on the pre-test and post-test using a paired t-test; significance was set at p<0.05. To find a 10% difference in post-test scores, a sample of 16 per group would be needed (**α**=0.05 and power of 0.80). Student comments were noted.

## RESULTS

Both methods of teaching led to improved knowledge, based on the pre- and post-test. The mean for the pre-test was 66% for the peer-taught group and 65% for the physician-taught group. The mean for the post-test was 75% for both groups (p=0.6). Both methods of simulation were viewed positively (Table). Participants in the peer-taught group agreed that student-directed learning was an effective way to learn. However, students in the physician-taught group thought their experience was better than those in the student-taught group (Table).

Student comments on the peer teaching included positive comments such as, “You really learn the case you are assigned much better than you would just reading about it;” “More interactive, at our learning level, fellow students understand better what may be difficult concepts;” “They were fun! (AND I learned a lot…) also it’s a more comfortable environment to ask fellow students questions…” But one student noted, “It’s just a personal preference that I tend to learn better from experts than students, but I didn’t mind participating in various modes of learning to accommodate all styles.”

## DISCUSSION

This study demonstrates student peer-taught simulations are both feasible and effective as a training tool during EM clerkships. Participants in peer-taught simulations achieved the same level of knowledge acquisition as those in physician-led sessions. However, students were not as satisfied with peer-taught simulation as with physician-led ones. There are advantages and disadvantages to peer-taught simulation.

Researchers have suggested a number of reasons why student-led teaching is effective.^8^ It is possible that peers explain ideas in a more relatable way that fellow students can easily understand. Students may feel more at ease asking questions of peers than of physicians. The act of teaching can also deepen the student instructor’s understanding of a topic.^9^ Although students have inherently less knowledge of the subject matter than do physicians, the act of teaching and the need for instructor understanding of a topic likely compensates for students’ lower expertise level and results in similar teaching and learning outcomes compared to physicians. It is also believed students would have improved retention of knowledge regarding the cases they taught, which several students commented on months later; this might be confirmed with further study.

There are additional advantages to student-led simulation. Perhaps the most important benefit of peer-assisted learning is introducing students to the art of clinical education. Teaching is an important aspect of being a competent physician. Indeed, mastering the ability to teach peers and patients is a competency required by many medical education accrediting organizations.^13^,^14^ Peer-assisted learning allows students to participate in clinical education in a way they may not have previously experienced, yet is a necessary skill as they transition into residency.

One significant drawback to small group teaching in simulation is the physician resources required. Peer-taught simulation reduces the amount of physician time required to perform the simulation training, providing a significant advantage over physician-led. Each month, our physician-led simulations required a total of nine hours of physician time, while our student-led simulations required only three hours for the single physician to monitor learning and to answer questions. We estimate that reduction in physician time results in an estimated annual reduction of 60 hours at an estimated cost savings of about $11,000. Thus, student-led simulation is time and cost efficient. Student teachers were also more likely to arrive on time and less likely to cancel compared to physician teachers in our study, which can save additional time and money by having more reliable training schedules and did not require a last-minute scramble to find a replacement. There is potential to use peer-led simulations on a broader scale. This model can be expanded to other areas of undergraduate medical education such as pre-clinical coursework or other clerkship rotations. Additionally, this model can allow for increased use of simulation-based learning for graduate medical education as well as for faculty development. Previous research has shown that simulation-based learning can be effective for both technical and non-technical skills attainment^2^ and can be used to teach skills such as teamwork or professionalism.^15^ Future studies should work to increase acceptability and study peer-teaching in other simulation settings.

## LIMITATIONS

There are important considerations that must be made regarding limitations of student-led simulation. Participants in the student-led groups viewed their experience less favorably than those in the physician-led groups. The limited research conducted on student perceptions of student-led teaching and what has been done is not in the area of simulation.^16^,^17^ A possible explanation of less favorable reactions is that students perceived that their peer teachers provided incomplete or insufficiently detailed information as compared to physician-led groups.^18^ In our study, a physician was present during the student-led simulations to answer questions, monitor what was being taught, correct mistakes, and to provide additional explanations. Since students did not experience both the physician-led and the student-led simulations, students in the student-led simulations may have been comparing their experience to a perceived experience with physicians that may not have been realistic. One concern for using student-led simulation is the perception that student-led simulation is “less good” than physician-led, and could affect their general perception of simulation in the future.

Another limitation of student-led simulations is that they create additional time demands for students, so tradeoffs may need to be made to ensure that students have adequate time to prepare. We hope the additional time leads to deeper understanding of key emergency care principles, but there was variability in engagement of the learner. Some learners would rather be told what they need to know instead of needing to spend out-of-classroom time to learn the information on their own. Our student-led teaching design also reduced opportunities to develop relationships between students and physicians, so it is important to ensure supplemental opportunities are available for students.

Further limitations include the possibility that students may have perceived student-led topics as being less valuable than physician-led topics, so adequate explanation must be given to ensure understanding of the importance of the topics being taught. The pre-test was given after the ACLS didactic on the rotation, but one confounder in regards to the post-test performance is that learning may have occurred on EM clinical shifts or via self-directed learning. We have submitted the cases to MedEdPortal.org so that others might reproduce or modify the study.

## CONCLUSION

In conclusion, student-led simulations are feasible, effective, time and cost efficient as a training tool during EM clerkships. However, while student-led simulation was viewed positively, students were not as satisfied with peer-taught simulations as with physician-taught ones. This study demonstrates the effectiveness of student-led simulations within EM clerkships, but also invites the possibility for broader use within medical education.

## Figures and Tables

**Table:** Student reactions to student-led and physician-led simulation teaching sessions (5-point scale).

Survey questions	Leader	Mean	SD	Sig*
(Leader)-directed learning was an effective way to learn new concepts	Physician	4.6	0.6	p=0.001
	Student	4.2	0.8	
(Leader)-directed modules will help me retain new concepts better	Physician	4.7	0.5	p<0.001
	Student	4.3	0.7	
I find (leader)-directed learning enjoyable	Physician	4.5	0.6	p=0.004
	Student	4.2	0.7	
I found the (leader)-directed learning more interactive than xxx-ran simulation	Physician	4.3	0.7	p=0.001
	Student	3.8	0.9	
I found the (leader)-directed learning more interactive than xxx-ran simulation	Physician	4.3	0.8	p<0.001
	Student	3.3	1.0	
I feel (leaders) were well prepared to run the simulation cases	Physician	4.7	0.7	p<0.001
	Student	4.3	0.6	
Overall, (leader)-directed simulation cases were a positive experience	Physician	4.7	0.5	p<0.001
	Student	4.3	0.7	
The simulation cases did not require too much additional work or time outside of this rotation	Physician	4.5	0.7	P=0.002
	Student	4.1	0.8	

* Stem questions are listed for the student-directed sessions, statistics analyzed with Mann-Whitney U.

*SD,* standard deviation
